# Adjuvant Radiation Therapy for Male Breast Cancer—A Rare Indication?

**DOI:** 10.3390/cancers12123645

**Published:** 2020-12-04

**Authors:** Tobias Forster, Clara Köhler, Rami El Shafie, Fabian Weykamp, Laila König, Nathalie Arians, Sebastian Adeberg, Laura Michel, Katharina Smetanay, Michael Golatta, Christof Sohn, Jörg Heil, Andreas Schneeweiss, Jürgen Debus, Juliane Hörner-Rieber

**Affiliations:** 1 Department of Radiation Oncology, Heidelberg University Hospital, 69120 Heidelberg, Germany; tobias.forster@med.uni-heidelberg.de (T.F.); koehler@stud.uni-heidelberg.de (C.K.); rami.elshafie@med.uni-heidelberg.de (R.E.S.); fabian.weykamp@med.uni-heidelberg.de (F.W.); laila.koenig@med.uni-heidelberg.de (L.K.); nathalie.arians@med.uni-heidelberg.de (N.A.); sebastian.adeberg@med.uni-heidelberg.de (S.A.); juergen.debus@med.uni-heidelberg.de (J.D.); 2 Heidelberg Institute of Radiation Oncology (HIRO), 69120 Heidelberg, Germany; 3 National Center for Tumor Diseases (NCT), 69120 Heidelberg, Germany; laura.michel@med.uni-heidelberg.de (L.M.); katharina.smetanay@med.uni-heidelberg.de (K.S.); andreas.schneeweiss@med.uni-heidelberg.de (A.S.); 4 Department of Medical Oncology, Heidelberg University Hospital, 69120 Heidelberg, Germany; 5 Department of Gynecology and Obstetrics, Heidelberg University Hospital, 69120 Heidelberg, Germany; michael.golatta@med.uni-heidelberg.de (M.G.); christof.sohn@med.uni-heidelberg.de (C.S.); joerg.heil@med.uni-heidelberg.de (J.H.); 6 German Cancer Research Center (DKFZ), 69120 Heidelberg, Germany; 7 Clinical Cooperation Unit Radiation Oncology, German Cancer Research Center (DKFZ), 69120 Heidelberg, Germany; 8 Heidelberg Ion-Beam Therapy Center (HIT), Department of Radiation Oncology, Heidelberg University Hospital, 69120 Heidelberg, Germany; 9 German Cancer Consortium (DKTK), Partner Site, 69120 Heidelberg, Germany

**Keywords:** male breast cancer, postoperative radiotherapy, local control, survival, toxicity

## Abstract

**Simple Summary:**

Male breast cancer (MBC) is a very rare disease and there are no randomized trials investigating the outcome of adjuvant radiotherapy in those breast cancer patients. Retrospective analysis is urgently needed to improve the evidence of adjuvant radiotherapy in male breast cancer. The study presents patient characteristics and survival outcomes of 41 consecutive male breast cancer patients treated with adjuvant radiotherapy of the chest wall or breast between 1990 and 2018. After a median follow-up of 80 months, the 5-year local control (LC) and locoregional control (LRC) rates were 100% and 97.4% (standard deviation (SD): 0.025), respectively. Five-year disease free survival (DFS) and overall survival (OS) rates were 64.6% (SD: 0.085) and 57.2% (SD: 0.082). No high-grade (Common Terminology Criteria for Adverse Events (CTCAE) grade > II) adverse events occurred after adjuvant radiotherapy. Our data provide a more scientific basis to assist clinicians with decision-making for adjuvant radiotherapy of male breast cancer patients.

**Abstract:**

Due to its rarity, there are no randomized trials investigating the outcome of adjuvant radiotherapy in MBC. This study reports on patient and tumor characteristics of 41 consecutive MBC patients treated between 1990 and 2018 and on clinical outcomes after surgical resection of tumors and adjuvant radiotherapy of the chest wall or breast. Local control (LC), locoregional control (LRC), overall survival (OS), disease-free survival (DFS), and toxicity were evaluated. After a median follow-up of 80 months (95% CI: 14.6–213.8 months) there was only one recurrence, in a patient’s locoregional lymph nodes 17 months after start of radiotherapy, resulting in an LC rate of 100% at 5 years and a 5-year LRC rate of 97.4% (standard deviation (SD): 0.025). Five-year DFS and OS rates were 64.6% (SD: 0.085) and 57.2% (SD: 0.082), respectively. Adjuvant radiotherapy was tolerated well without high-grade (CTCAE grade > II) adverse events. After tumor resection and adjuvant radiotherapy, LC and LRC rates in MBC patients are excellent and comparable to results found for female breast cancer (FBC) patients. However, as patients are often diagnosed with locally advanced, higher-risk tumors, distant recurrences remain the major failure pattern.

## 1. Introduction

Representing 1% of all breast tumors worldwide, male breast cancer (MBC) is a rare disease [1]. For MBC, there are no randomized trials investigating the outcome of adjuvant radiotherapy, and most clinical trials on breast cancer have routinely excluded men [2]. However, epidemiological studies indicate that the incidence of MBC is increasing, at least partially due to improved awareness and earlier detection [3,4]. MBC is commonly detected by the appearance of a painless retroareolar mass, and most men are at an advanced stage with high rates of lymph node involvement at the time of diagnosis [2,5]. There are also other presentations of MBC, including ulceration, retraction, nipple bleeding, or presentation as an abscess [6]. Known risk factors are mutations in tumor-suppressor genes like BRCA2 (breast cancer 2) and a positive family history [7]. In comparison to female breast cancer (FBC), a higher prevalence of estrogen-receptor positivity and only a 9% rate of HER2 (human epidermal growth factor receptor 2)-positive tumors is reported in the literature for MBC, with 10% of cases presenting as ductal carcinoma in situ (DCIS) [8]. Modern treatment strategies and information regarding the oncologic outcome are mainly based on small retrospective studies or are translated from breast cancer studies with female participants. Due to the lack of evidence for this rare disease, MBC is treated similarly to female breast cancer (FBC), including surgical resection, adjuvant radiotherapy, chemotherapy, and endocrine therapy [9]. In order to reach sufficient margins in male breast tissue, modified radical mastectomy is the most common surgical procedure for the treatment of MBC [4,10,11]. Although the evidence for radiotherapy is limited for MBC, current guidelines recommend adjuvant irradiation for tumor stage ≥ T2, for negative hormone receptor status, and in cases where axillary lymph nodes are involved [9]. These recommendations are based on some studies indicating an improved outcome after adjuvant radiation therapy of the chest wall [11,12,13,14]. While the prognosis for MBC seems to have improved over time due to earlier detection, data regarding the prognosis in comparison to FBC are inconsistent [15]. However, adjusted by age, stage, and histology, comparable outcomes have been reported in the literature [16,17,18,19]. The outcome of a first collective of 25 MBC patients who received radiotherapy to the chest wall between 1981 and 2000 at Heidelberg University Hospital was published in 2005 by Zabel et al. [20]. It is the aim of the present analysis to describe patient and tumor characteristics of MBC patients treated at the Department of Radiation Oncology at Heidelberg University Hospital over more than two decades (1990–2018) and to report on the clinical outcomes after surgical resection and adjuvant radiotherapy. Data from eight MBC patients from the prior analysis of Zabel et al., treated between 1990 and 2000, has been updated and included in this publication.

## 2. Results

### 2.1. Patient and Tumor Characteristics

In total, 41 consecutive patients with MBC treated with postoperative external beam radiotherapy of the chest wall or the breast (8 patients received 2D conventional radiotherapy, 25 patients 3D conventional radiotherapy, and 8 patients intensity-modulated radiotherapy (IMRT)) between September 1990 and October 2018 were included in this analysis. Patient, tumor, and treatment characteristics are illustrated in Table 1. Median patient age at the time of diagnosis of MBC was 67 years. Thirty-four percent (*N* = 14) of the patients had a family history of cancer, while 15% (*N* = 6) did not. For 21 (51%) men, history of cancer in their family was unknown. The most prevalent histology of MBC was invasive ductal carcinoma (83%; *N* = 34), followed by DCIS (5%; *N* = 2), mainly with a central location (83%; *N* = 34). None of the patients had distant metastases at the time of diagnosis. Tumors were staged pTis to pT4c, with 90% of tumors ≥pT1c. In total, the average tumor size was 2.6 cm. After tumor resection (mastectomy: *N* = 38 or local excision: *N* = 3), two patients had positive resection margins after radical mastectomy (one R1 and one R2), while all other patients were resected R0. Most tumors were staged G2–3 (95%; *N* = 39), were estrogen- and progesterone-receptor-positive (85%; *N* = 35 and 80%; *N* = 33, respectively), and were HER2-negative (71%; *N* = 29). Sixty-one percent (*N* = 25) of patients had a positive histological lymph node status at the time of diagnosis and 85% (*N* = 35) of patients received axillary lymph node dissection, while sentinel lymph node biopsy was performed in 17% (*N* = 7) of cases. Three patients received axillary lymph node dissection due to a positive sentinel lymph node biopsy. Surgical resection of lymph nodes was not performed for two patients with non-invasive MBC. Overall, a mean of 13 lymph nodes (range 1–41) were removed. Radiotherapy of the breast or chest wall was performed with a total dose of 40.05 to 50.4 Gy in fractions of 1.8 to 2.67 Gy and an additional boost irradiation of 10–16 Gy was performed in 24 patients. Due to axillary lymph node metastases, twenty-three (56%) patients received additional irradiation of the lymphatic drainage with the same total dose and number of fractions. Chemotherapy was administered in 18 (44%) cases, and 81% (*N* = 33) of patients received endocrine therapy. Most patients receiving adjuvant chemotherapy were treated with EC including epirubicin and cyclophosphamide; FEC including 5-fluorouracil, epirubicin, and cyclophosphamide; ETC including epirubicin, paclitaxel, and cyclophosphamide; or with chemotherapy including docetaxel and cyclophosphamide. In two patients with HER2/neu-positive tumors, trastuzumab was additionally administered. One patient received neoadjuvant chemotherapy with docetaxel in combination with anti-HER2 treatment (trastuzumab and pertuzumab). Endocrine therapy consisted of tamoxifen in 29 cases, while 4 patients were treated with aromatase inhibitors.

### 2.2. Oncological Outcome

**Figure 1:**
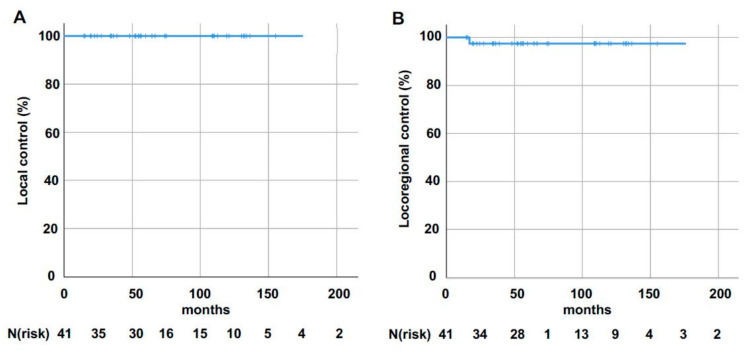
Kaplan–Meier-estimated local (**A**) and locoregional (**B**) control following surgical resection and postoperative radiotherapy for male breast cancer (MBC).

Median follow-up for all patients was 79.7 months after the start of radiotherapy (95% confidence interval (CI): 14.6–213.8 months). None of the MBC patients developed local tumor recurrence during the follow-up period, leading to a 10-year Kaplan–Meier-estimated local control (LC) rate of 100% (Figure 1A). In terms of locoregional control, the Kaplan–Meier-estimated locoregional control (LRC) rate was 97.4% (standard deviation (SD): 0.025) at 10 years, with one patient suffering from recurrence in regional lymph nodes 17 months after start of radiotherapy (Figure 1B). The patient was initially staged pT1cN3a G2. The tumor was hormone receptor positive and HER2 negative. Mastectomy and axillary lymph node dissection (12 involved nodes out of 32) were performed, followed by adjuvant irradiation of the chest wall and lymphatic drainage (supra- and infraclavicular fossa, and lymphatic drainage of the internal mammary artery). Adjuvant systemic therapy and endocrine therapy were administered using epirubicin, paclitaxel, cyclophosphamide, and tamoxifen.

**Figure 2:**
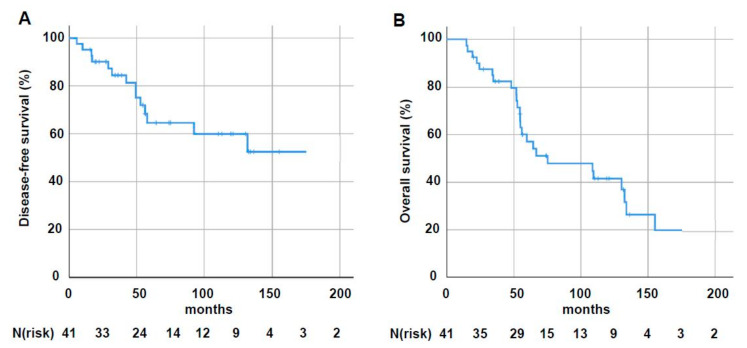
Kaplan–Meier-estimated disease-free (**A**) and overall (**B**) survival following surgical resection and postoperative radiotherapy for MBC.

The Kaplan–Meier-estimated disease-free survival (DFS) rate was 64.6% (SD: 0.085) at 5 years and 60% (SD: 0.09) at 10 years, respectively (Figure 2A). Fourteen patients (34%) developed distant metastases during follow-up: bone metastases in nine (22%) cases and metastases of the lung in six patients (15%) were most prevalent, followed by metastases of the brain (10%; *N* = 4), non-regional lymph nodes (10%, *N* = 4), the liver (2%; *N* = 1), and the skin (2%; *N* = 1). Distant metastases were treated with systemic therapy, radiotherapy, and surgery in 17% (*N* = 7), 17% (*N* = 7), and 5% (*N* = 2) of cases, respectively. Regarding overall survival (OS), the Kaplan–Meier-estimated rate was 57.2% (SD: 0.082) at 5 years and 41.6% (SD: 0.084) at 10 years, respectively (Figure 2B). In univariate analysis, no significant impact of any of the tested potential risk factors (age, BMI, grade, tumor size, nodal status, nodal irradiation, chemotherapy) on OS, DFS, or LRC was detected.

### 2.3. Toxicity

Postoperative radiotherapy was tolerated well, with only mild acute toxicity. Results for acute toxicity and late side effects of treatment are illustrated in Table 2. The most prevalent acute side effects were cutaneous erythema and dry desquamation with Common Terminology Criteria for Adverse Events (CTCAE) grades I–II in 90% of cases (*N* = 37). Nine patients (22%) developed treatment related fatigue CTCAE grades I–II, while mild pain (CTCAE grade I) was reported in three cases (7%) after radiotherapy. No further acute toxicities or any side effects CTCAE grades III–V were detected.

In terms of late toxicity, 56% of patients developed side effects of CTCAE grade I, with hyperpigmentation seen in 39% (16/41) of patients, lymphedema in 24% (10/41), restriction of arm movement in 10% (4/41), and telangiectasia in 2% (1/41), respectively. There was no late toxicity higher than CTCAE grade I detected in the present analysis. During follow-up, six patients were diagnosed with further malignancies. Three patients suffered from prostate cancer, one patient from hepatocellular carcinoma, and two patients developed lung cancers.

## 3. Discussion

Strategies for the treatment of MBC are derived from clinical experiences in FBC, for which multiple randomized controlled trials exist, demonstrating a significant benefit of adjuvant radiotherapy on local control and overall survival after breast-conserving surgery (BCS) or mastectomy [21,22,23]. Current guidelines still lack randomized trials for MBC to enable recommendations for postoperative radiotherapy in men on a more scientific basis [2,24]. However, retrospective studies of MBC emphasize the improvement in LC and OS after adjuvant radiotherapy [25]. In men, benefits of postoperative radiotherapy have been seen for both early stages and locally advanced stages of breast cancer, with the involvement of lymph nodes or stage III disease [13,14,25,26]. While an increase in the application of postoperative radiotherapy after mastectomy in male breast cancer patients has been detected over the last decades, adjuvant radiotherapy is still underutilized in men, especially when compared to clinical practice in female patients [17,27,28]. After BCS, only 42% of men with stage I breast cancer were administered postoperative radiotherapy, based on an analysis of the Surveillance Epidemiology and End Results (SEER) database [29]. MBC patients with high-risk features, such as tumor stage T2 or higher, negative hormone receptor status, and involvement of axillary lymph nodes, are more likely to receive postoperative radiotherapy [9]. Scott-Conner et al. analyzed stage-specific discrepancies in the treatment of comparable breast cancer cases of both sexes and reported preferential application of adjuvant radiotherapy in high-risk male breast cancer patients, while men with low-risk breast cancer were less likely to receive radiotherapy after lumpectomy as compared to women [30]. Due to the lack of randomized trials for MBC assessing the impact of radiotherapy, and owing to the fact that historically most studies for MBC were underpowered due to a small sample size, it has been challenging to detect significant survival benefits of adjuvant irradiation [31,32,33]. In the current study, rates for LC and LRC were comparable to results reported in the literature for women with breast cancer after mastectomy and postoperative radiotherapy [34]. The presented LC and LRC rates were also in line with the data of other authors evaluating the outcome of MBC patients and with the older analysis of Zabel et al. [20,35,36].

With respect to DFS, 5-year rates of 91% to 98% and 10-year rates of 81% are presented in the literature for early-stage FBC after BCS and postoperative radiotherapy [21,37,38]. Contrary to these results, in the current study DFS rates were 65% and 60% after 5 and 10 years for MBC, respectively. Similar results for DFS in MBC patients were reported by others [11,16,20,36]. This discrepancy between sexes may be explained by the patient characteristics: as shown in other studies, the prognosis of men with breast cancer is predicted by factors including tumor stage, nodal involvement, histological grade, and age at diagnosis [5,27,39,40]. In this study, most men presented with tumors ≥pT2, node-positive disease, and were graded G2 or G3. When compared to high-risk female breast cancer patients with node-positive disease who received mastectomy and adjuvant radiotherapy, DFS rates of 60% are in line with results from female patients [22,34]. Our results for DFS have been confirmed by others evaluating the outcome of MBC patients after postoperative radiation therapy in retrospective studies and reporting 5-year DFS rates of 53% to 66% [11,16,36].

Contrary to our results in men (5-year OS of 57%), 5-year OS rates of >70% are reported following BCS and adjuvant radiotherapy in women with node-positive breast cancer [21,22]. In another randomized trial, 5-year OS rates of 84% and 10-year rates of 67% were presented for female breast cancer patients staged pT3N0 after mastectomy and postoperative radiotherapy [41]. Our OS data also differ from the results of other studies evaluating the outcome of postoperative radiotherapy in male breast cancer patients. In the literature, a broad range of 5-year OS rates of 67% to 96% is reported for men after adjuvant radiotherapy, with the most favorable OS presented in the analyses of Rogowski et al. and Rolf et al. [11,14,16,18,35,36]. While in the observational study of Rogowski et al. an OS rate of 88% was reported at 5 years for MBC patients after postoperative radiotherapy, Rolf et al. presented a 5-year rate of 96% in their analysis [35,36]. Both values differ significantly from the results of our study, where a 5-year OS rate of only 57% was seen. Contrary to the analysis of Rogowski et al., there were also patients with HER2/neu-positive and hormone-receptor-negative tumors in our cohort and only 5% of patients were graded G1 in our study, compared to 16% in the trial of Rogowski et al. However, the small sample size of 21 irradiated patients in the study of Rogowski et al. may explain the different OS results [35]. In comparison to Rolf et al., there were also differences in patient characteristics, with 96% estrogen-receptor-positive tumors and 90% progesterone-positive tumors in their study, compared to 85% and 80% positive tumors seen in our analysis, respectively. While 18% of their MBC patients were graded G3, 29% of cases were graded G3 in our trial. Furthermore, our study cohort consisted of fewer patients with stage I disease (7% versus 21%) and more men with stage III tumors (49% versus 24%) [36]. More favorable baseline characteristics are also seen in other publications for MBC, which report more favorable OS rates after postoperative radiotherapy [11,14]. In line with our results, other studies demonstrated more similar OS results [16,18] and this study’s OS rates are also consistent when compared to those of the older analysis of Zabel et al. [20].

Considering the discrepancy between excellent LC and limited OS and DFS due to predominant distant failure, seen in the present study and in others, one could question the role of post-mastectomy radiation in MBC. However, based on the present data, it is difficult to decide whether excellent LC was based on postoperative radiotherapy; MBC patients might have good LC rates even without radiation. Several studies by other authors tried to address this issue: in a retrospective analysis of Yu et al., 81 MBC patients were evaluated to compare the outcome of patients treated with post-mastectomy radiation to patients who received mastectomy alone. In their trial a significantly better LC was demonstrated for patients treated with radiotherapy, but no benefit in OS was observed [11]. In population-based studies, Sroufe et al. and Abrams et al. also failed to demonstrate a survival benefit of post-mastectomy radiation for MBC patients [14,18].

In total, adjuvant radiotherapy was well-tolerated with only mild side effects in the current study. Our toxicity results are in line with more recent studies, which report mainly skin-related reactions without the occurrence of grade III to V toxicities. Reported late side effects also did not differ from those in the literature [36].

One limitation of the presented single-center study is its small sample size due to the rarity of MBC. However, consecutive patients treated during nearly three decades were included to enlarge the patient numbers and quality of data. During this long period of time, diagnostic approaches and irradiation techniques changed several times to include 2D conventional radiotherapy, 3D conventional radiotherapy, or intensity-modulated radiotherapy (IMRT), with different dose prescriptions and distributions. The increasing influence of biological markers during these decades also changed indications, as did the decision-making for postoperative radiotherapy, leading to a very heterogeneous cohort. The retrospective design is also a limiting factor, but randomized clinical trials are unlikely to be conducted for this rare disease. The present data do not provide evidence to support or refute the role of postoperative radiotherapy in MBC. Nevertheless, the presented analysis contributes to the small amount of existing evidence for patient and tumor characteristics of MBC, and the outcome and the pattern of treatment for this rare disease are of crucial interest to the scientific community.

## 4. Materials and Methods

### 4.1. Patients and Treatment

Treatments of patients were discussed interdisciplinarily by gynecologists, medical oncologists, radiologists, pathologists, and radiation oncologists. Before the start of treatment, patients were staged for locoregional and distant metastases according to guidelines at the time of treatment. According to current guidelines, surgical resection with sentinel lymph node biopsy or axillary lymph node dissection was performed, followed by external beam radiation therapy (EBRT) of the chest wall or the breast with or without regional nodal irradiation, depending on the pathological axillary lymph node status. Depending on the year of treatment, the respective patient received radiotherapy according to the state of the art, including 2D conventional radiotherapy, 3D conventional radiotherapy, and IMRT. The total treatment dose was 40.05–50.4 Gy and single fraction doses of 1.8–2.67 Gy followed by either a 10–16 Gy boost or no boost. Treatment characteristics are listed in Table 1. Systemic adjuvant treatment was administered according to national guidelines at the time of treatment.

### 4.2. Statistical Analysis

Patient data were retrieved retrospectively from institutional databases in accordance with institutional ethical policies. Analyses of surgical and pathological reports as well as reviews of treatment plans were performed. Overall survival (OS) was calculated in months from the start of radiotherapy until the last date of follow-up or death. Disease-free survival (DFS), as well as local control (LC) and locoregional control (LRC), were calculated from the start of radiotherapy until the first diagnosis of recurrent disease. Local recurrence was defined as any relapse within the ipsilateral breast tissue, while locoregional recurrence was regarded as occurrence of regional lymph node metastases (axilla, supra- and infraclavicular fossa, internal mammary chain). Recurrences at any other sites were classified as distant metastases. The data analysis was censored, as not all patients suffered from an event during follow-up time.

The Kaplan–Meier method was used to estimate survival rates of OS, DFS, LC, and LRC. Potential risk factors (age, body mass index, grade, tumor size, nodal status, nodal irradiation, chemotherapy) were tested in a univariate analysis (logrank test) to detect the impact on OS, DFS, and LRC. Statistical analysis was performed using the software tool SPSS 24.0 (IBM, Armonk, NY, USA). Acute toxicity was defined as occurrence of side effects within three months following radiotherapy, while later occurrence was classified as late toxicity. Toxicity was evaluated according the Common Terminology Criteria for Adverse Events (CTCAE) version 5.0.

### 4.3. Ethical Approval

The analysis was approved by the Ethics Committee of Heidelberg University (S-757/2019).

## 5. Conclusions

After tumor resection and adjuvant radiotherapy in patients with MBC, LC and LRC rates were excellent and comparable to the rates of female breast cancer patients. Adjuvant radiotherapy was well-tolerated, with only mild acute toxicity and late side effects. The comparably high rate of distant recurrences underlines the need for early aggressive systemic treatment in this high-risk patient subgroup.

## Figures and Tables

**Table 1:** Characteristics of patients, tumor, and treatment.

Characteristics		Patients (Total: 41)	%
**Patient Characteristics**			
Age at diagnosis	Median (years)	67	
	Range (years)	36–83	
BMI	<30	23	56.1
	30–34.9	5	12.2
	>34.9	3	7.3
	unknown	10	24.4
Performance status (Karnofsky)	Median (%)	90	
	Range (%)	60–100	
**Tumor Characteristics**			
Histology	DCIS	2	4.9
	Invasive ductal	34	82.9
	Invasive lobular	3	7.3
	Other	2	4.9
Side	Right breast	19	46.3
	Left breast	22	53.7
Location	Lower inner	1	2.4
	Lower outer	0	0
	Upper inner	0	0
	Upper outer	6	14.7
	Central	34	82.9
Tumor size	Median (cm)	2.6	
	Range (cm)	0.6–5.5	
R status	R0	39	95.2
	R1	1	2.4
	R2	1	2.4
pT status	pTis	2	4.9
	pT1a	1	2.4
	pT1b	1	2.4
	pT1c	8	19.5
	pT2	17	41.6
	pT3	1	2.4
	pT4a	1	2.4
	pT4b	9	22.0
	pT4c	1	2.4
pN status	pN0	16	39.0
	pN1	13	31.7
	pN2	7	17.1
	pN3	5	12.2
Lymphangiosis	Yes	19	46.3
	No	13	31.7
	Unknown	9	22.0
Tumor grade	G1	2	4.9
	G2	27	65.9
	G3	12	29.2
Estrogen receptor status	Positive	35	85.3
	Negative	2	4.9
	Not tested	4	9.8
Progesterone receptor status	Positive	33	80.4
	Negative	4	9.8
	Not tested	4	9.8
HER2/neu status	Positive	2	4.9
	Negative	29	70.7
	Not tested	10	24.4
Ki-67 status	Median (%)	19.0	
	Range (%)	5–50	
**Treatment Characteristics**			
Surgical procedure	Mastectomy	38	92.7
	BCS	3	7.3
Axillary dissection	Yes	35	85.4
	No	6	14.6
Sentinel lymph node extirpation	Yes	7	17.1
	No	34	82.9
No. of resected lymph nodes	Median	13	
	Range	1–41	
Chemotherapy	None	23	56.1
	Neoadjuvant only	0	0
	Adjuvant only	17	41.5
	Neoadjuvant and adjuvant	1	2.4
Endocrine therapy	Yes	33	80.5
	No	8	19.5
Radiation technique	IMRT	8	19.5
	3D-CRT	25	61.0
	2D-CRT	8	19.5
Nodal irradiation	Yes	23	56.1
	No	18	43.9
	Irradiation of IMA	8	19.5
	Irradiation of SCN	19	46.3
	Irradiation of ALN	7	17.1
Treatment dose	Median (Gy)	50.0	
	Range (Gy)	40.05–50.4	
Single fraction dose	Median (Gy)	1.9	
	Range (Gy)	1.8–2.67	
Boost	Yes	24	58.5
	No	17	41.5
Boost dose	Median (Gy)	10	
	Range (Gy)	10–16	

Estrogen receptor positive: ≥1% of cells stained for estrogen receptor; Progesterone receptor positive: ≥1% of cells stained for progesterone receptor; HER2/neu positive: ≥3% by immunohistochemistry or gene amplification by fluorescence in situ hybridization. Abbreviations: No.: number; Gy: Gray; BCS: breast-conserving surgery; IMRT: intensity-modulated radiotherapy; 3D-CRT: 3-dimensional conventional radiotherapy; 2D-CRT: 2-dimensional conventional radiotherapy; IMA: internal mammary artery lymph nodes; SCN: supraclavicular lymph nodes; ALN: axillary lymph nodes; DCIS: ductal carcinoma in situ.

**Table 2:** Acute toxicity and late side effects of treatment.

Toxicity		Patients (Total: 41)	%
**Acute Toxicity**		38	92.7
Pain	Any grade	3	7.3
	CTCAE grade I	3	7.3
	CTCAE grade ≥ II	0	0
Fatigue	Any grade	9	21.9
	CTCAE grade I	8	19.5
	CTCAE grade II	1	2.4
	CTCAE grade III	0	0
Erythema and dry desquamation	Any grade	37	90.2
	CTCAE grade I	24	58.5
	CTCAE grade II	13	31.7
	CTCAE grade III	0	0
**Late Side Effects**		23	56.1
Hyperpigmentation	CTCAE grade I	16	39.0
	CTCAE grade ≥ II	0	0
Lymphedema	CTCAE grade I	10	24.4
	CTCAE grade ≥ II	0	0
Fibrosis	Any grade	0	0
Telangiectasia	CTCAE grade I	1	2.4
	CTCAE grade ≥ II	0	0
Restriction of arm movement	CTCAE grade I	4	9.8
	CTCAE grade ≥ II	0	0

Abbreviations: CTCAE: Common Terminology Criteria for Adverse Events.

## Abbreviations

BCS:	Breast-Conserving Surgery
BRCA2:	Breast Cancer 2 Gene
CI:	Confidence Interval
DCIS:	Ductal Carcinoma In Situ
DFS:	Disease-Free Survival
EBRT:	External Beam Radiation Therapy
FBC:	Female Breast Cancer
HER2:	Human Epidermal Growth Factor Receptor 2
HER2/neu:	Human Epidermal Growth Factor Receptor 2
IMRT:	Intensity-Modulated Radiotherapy
LC:	Local Control
LRC:	Locoregional Control
MBC:	Male Breast Cancer
OS:	Overall survival
SD	Standard Deviation
